# Roles of N6-Methyladenosine (m^6^A) in Stem Cell Fate Decisions and Early Embryonic Development in Mammals

**DOI:** 10.3389/fcell.2020.00782

**Published:** 2020-08-11

**Authors:** Meng Zhang, Yanhui Zhai, Sheng Zhang, Xiangpeng Dai, Ziyi Li

**Affiliations:** Key Laboratory of Organ Regeneration and Transplantation of Ministry of Education, First Hospital, Jilin University, Changchun, China

**Keywords:** N6-methyladenosine, RNA metabolism, stem cell fate, cell reprogramming, embryonic development

## Abstract

N6-methyladenosine (m^6^A) is one of the most abundant internal mRNA modifications, and it affects multiple biological processes related to eukaryotic mRNA. The majority of m^6^A sites are located in stop codons and 3′UTR regions of mRNAs. m^6^A regulates RNA metabolism, including alternative splicing (AS), alternative polyadenylation (APA), mRNA export, decay, stabilization, and translation. The m^6^A metabolic pathway is regulated by a series of m^6^A writers, erasers and readers. Recent studies indicate that m^6^A is essential for the regulation of gene expression, tumor formation, stem cell fate, gametogenesis, and animal development. In this systematic review, we summarized the recent advances in newly identified m^6^A effectors and the effects of m^6^A on RNA metabolism. Subsequently, we reviewed the functional roles of RNA m^6^A modification in diverse cellular bioprocesses, such as stem cell fate decisions, cell reprogramming and early embryonic development, and we discussed the potential of m^6^A modification to be applied to regenerative medicine, disease treatment, organ transplantation, and animal reproduction.

## Introduction

Since the discovery of the first structurally modified nucleoside, pseudouridine, in the 1950s (Cohn and Volkin, 1951), more than 150 kinds of chemical modifications have been found on cellular RNA (Boccaletto et al., 2018). Due to the recognition of the prevalence and functional significance of N6-methyladenosine (m^6^A) modification on mRNA as well as the development of high-throughput sequencing technologies, there has been recent widespread interest in the biological phenomena of RNA modification (Dominissini et al., 2012; Meyer et al., 2012). In 1974, m^6^A was first discovered as the major form of internal methylation of mammalian mRNA (Desrosiers et al., 1974; Perry and Kelley, 1974). Early studies indicated that m^6^A occurred in the (G/A; m^6^A) C sequences of RNA and was predominately enriched in the stop codons and 3′ untranslated regions (3′UTRs; Schibler et al., 1977; Wei and Moss, 1977). m^6^A modifications are added or removed by a series of methyltransferases (also known as writers) and demethylases (also known as erasers; Jia et al., 2011; Liu et al., 2013; Zhen et al., 2013; Ping et al., 2014). In addition, the m^6^A site is recognized by binding proteins (also known as readers; Wang X. et al., 2014; Wang et al., 2015; Xiao et al., 2016; Li et al., 2017; Mao et al., 2019). With the development of global-wide m^6^A detection technology, epitranscriptome data have revealed large amounts of transcripts across various species and tissues in normal and pathological processes (Dominissini et al., 2012; Meyer et al., 2012; Huang et al., 2019). It is widely believed that DNA and histone modifications play crucial roles in gene expression regulation (Egger et al., 2004). However, a series of recent studies have shown that m^6^A has notable effects on the regulation of gene expression at the post-transcriptional level, animal development, and human diseases (Wang Y. et al., 2014; Wang et al., 2015; Roundtree et al., 2017a).

In this review, we summarized the latest progress regarding the molecular basis of m^6^A effectors and discussed the functional roles of m^6^A modification in the regulation of RNA metabolism. In addition, we focused on the molecular regulation mechanism of m^6^A modification in stem cell fate decisions and early embryonic development. Our review may contribute to a better understanding of RNA modification and its mechanism in the life sciences area.

## Overview of RNA m^6^A Modification

### Characteristics of m^6^A Modification and Related Enzymes

**FIGURE 1:**
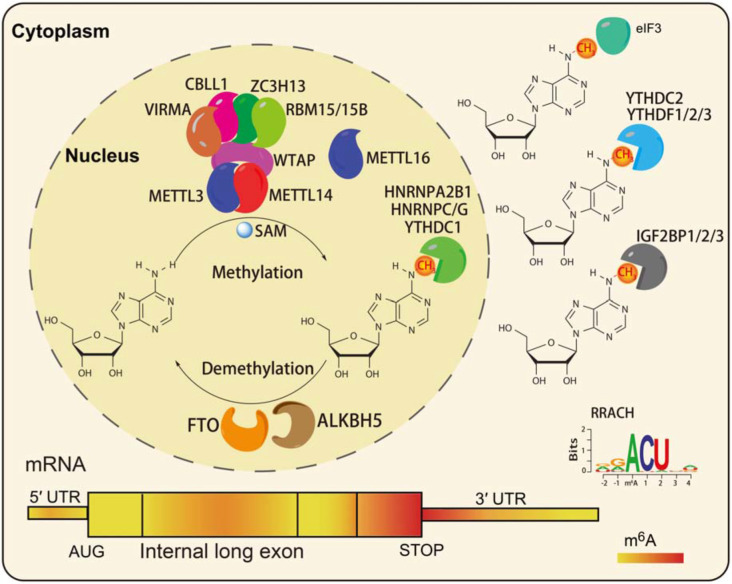
The characteristics of RNA m^6^A modification. The m^6^A writers, erasers, and readers in eukaryotic cells (Shi et al., 2019). The preferences and density of RNA m^6^A modification in different regions of mRNAs (Fitzsimmons and Batista, 2019).

N6-methyladenosine modifications are highly species-conserved between yeast, plants, fruit flies, and mammals. Recent transcriptome-wide m^6^A site positioning has provided more details about its location and prominence, revealing its universality among thousands of transcripts in humans and mice (Dominissini et al., 2012). m^6^A modifications mainly occur on adenine (A) in the RRACH (R = G or A, G > A, H = A or C or U, and U > A > C) sequence, and is mainly located near the stop codons and 3′UTR of mRNAs (Dominissini et al., 2012; Meyer et al., 2012; Figure 1). In mammals, approximately 0.1 to 0.6% of adenines undergo m^6^A modification, with an average of 3 to 5 methylated sites in each mRNA. Notably, m^6^A modifications can be deposited onto the transcripts in tissue- and cell-type-specific manners (An et al., 2020).

Similar to DNA methylation, the m^6^A levels in RNA are dynamic and reversible. A series of m^6^A readers make up the methyltransferase complex (MTC), including the core component methyltransferase-like 3 (METTL3), and methyltransferase-like 14 (METTL14; Liu et al., 2013), and other regulatory factors: Wilms tumor 1-associating protein (WTAP; Ping et al., 2014), Vir like m^6^A methyltransferase associated (VIRMA; Yue et al., 2018), zinc finger CCCH-type containing 13 (ZC3H13; Wen et al., 2018), Cas-Br-M (murine) ecotropic retroviral transforming sequence-like 1 (CBLL1), and RNA binding motif protein (RBM15/15B; Patil et al., 2016). METTL3 is the main catalytic subunit of MTC, METTL14 mainly promotes binding to RNA, and WTAP is the regulatory subunit, which binds to METTL3/14 and thus contributes to the catalytic activity of methyltransferase and the deposition of m^6^A (Ping et al., 2014). Recent studies reported that zinc finger CCHC-type-containing 4 (ZCCHC4) and methyltransferase-like 16 (METTL16) function as m^6^A methyltransferases of 28S rRNA and U6 snRNA, respectively (Brown et al., 2016; Pendleton et al., 2017; Ma et al., 2019). Fat mass and obesity-associated protein (FTO) and alkB homolog 5 (ALKBH5) have been identified as m^6^A demethylases that play function in an Fe (II) and α-ketoglutarate (αKG)-dependent manner (Jia et al., 2011; Zhen et al., 2013). FTO was the first identified demethylase, and it can remove the m^6^A modification in the internal (m^6^A) and 5′cap (m^6^A_m_) of mRNAs in different environments (Wei et al., 2018).

To date, three classes of m^6^A readers have been characterized: the first class, YT521-B homology (YTH)-domain containing proteins, directly bind m^6^A-modified mRNAs (Wang X. et al., 2014; Wang et al., 2015; Li et al., 2017; Shi et al., 2017; Figure 1); the second class, RNA structure-dependent proteins, include HNRNPC/G, and HNRNPA2B1 (Liu et al., 2015, 2017; Alarcon et al., 2015b; Figure 1); the third class, RNA binding proteins such as insulin-like growth factor 2 mRNA-binding proteins 1–3 (IGF2BP1/2/3), and Fragile X mental retardation protein (FMRP), can bind m^6^A-modified mRNAs through RNA binding domains (RBDs), such as K homology (KH), RNA recognition motif (RRM), and arginine/glycine-rich (RGG) domains (Huang et al., 2018; Edens et al., 2019; Figure 1). A recent study reported that a neuronal cell-specific m^6^A reader proline rich coiled-coil 2 A (Prrc2a), which contains glycine, arginine and glutamic acid (GRE) domains, played an important role in oligodendroglial specification and myelination (Wu et al., 2019b).

Writers and erasers contribute to the establishment, maintenance, and remodeling of the global m^6^A modification state across various species, tissues and cells in normal, and pathological processes. The subcellular localization of m^6^A effectors and regulators is essential for their function. METTL3 and YTHDF2 may depend on their cellular location and expression to show multiple functions (Zhou et al., 2015; Lin et al., 2016). The demethylase FTO shows different substrate preferences in the cytoplasm and in the nucleus (Wei et al., 2018). Notably, the m^6^A modification-related methylase can be regulated by various factors involved in transcriptional, posttranscriptional (miRNAs and lncRNAs), and translational (phosphorylation, ubiquitination, or SUMOylation modification) regulation (Du et al., 2018; Schöller et al., 2018; Zhu et al., 2018). In addition, several m^6^A modification regulators, such as ZFP217, SMAD2/3, CEBPZ, TARBP2, and TRA2A, have been found to regulate m^6^A modification of mRNAs by recruiting or repelling MTC in a cell-type-specific manner (Aguilo et al., 2015; Barbieri et al., 2017; Bertero et al., 2018; Fish et al., 2019; An et al., 2020). In addition, a recent study reported that H3K36me3 recruited METTL14 thus guiding m^6^A deposition on nascent transcripts (Huang et al., 2019). Liu et al. (2020) recently revealed that m^6^A of chromosome-associated regulatory RNAs (carRNAs) can regulate chromatin state and transcription through increasing activate histone modifications, such as H3K4me3 and H3K27ac. In embryonic neural stem cells (eNSCs), METTL14 knockout (KO) increases H3K4me3, H3K27me3, and H3K27ac levels by affecting the mRNA stabilization of histone-modifying enzymes (Wang et al., 2018). These results suggest that there is crosstalk between these diverse epigenetic modifications, which results in the regulation of gene expression.

### The Effects of m^6^A on RNA Metabolism

**FIGURE 2:**
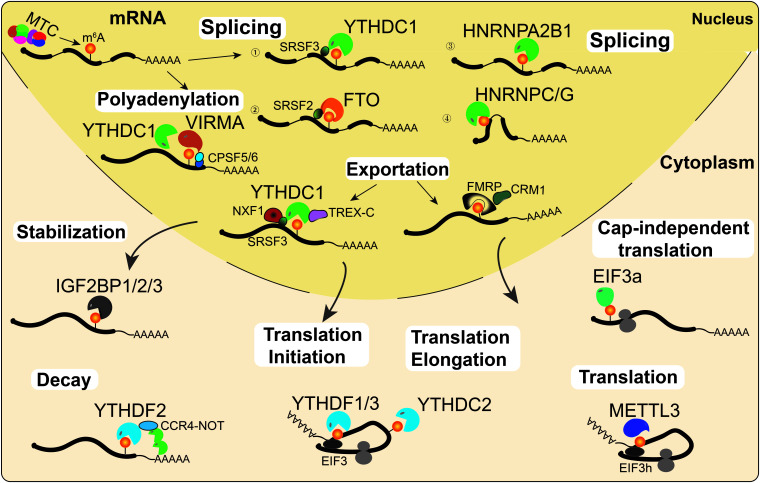
RNA m^6^A modifications regulate RNA metabolism. m^6^A is deposited onto nascent mRNAs by MTC. Then the m^6^A-modified mRNAs undergo AS by recruiting splicing factors to m^6^A sites or their flanking sequences. On the other hand, YTHDC1 and VIRMA can interact with the polyadenylation cleavage factors CPSF5 and CPSF6, thus regulating mRNA APA. Then, mRNAs can be recognized by YTHDC1 or FMRP and exported into the cytoplasm. Cytoplasmic m^6^A readers regulate mRNA stability (IGF2BPs), decay (YTHDF2), and translation (YTHDF1/3, YTHDC2, EIF3a, and METTL3) under normal and stress conditions.

Gene expression is regulated by multiple processes, including transcription, post-transcriptional regulation, and translation. An increasing number of studies have shown that RNA m^6^A modification affects RNA processing and metabolism, including alternative splicing (AS), alternative polyadenylation (APA), mRNA stability, export, degradation, and translation (Figure 2).

#### m^6^A Affects Pre-mRNA Splicing, Polyadenylation and Export

The majority of m^6^A writers and erasers are located in nuclear speckles, suggesting that they may regulate RNA processing (Liu et al., 2013; Ping et al., 2014; Yue et al., 2018). Indeed, increasing evidence suggests that m^6^A is associated with AS and APA events (Zhen et al., 2013; Xu et al., 2017). In HeLa cells, PAR-CLIP data analysis suggested that METTL3 and WTAP affected mRNA AS and the expression of genes related to transcription and RNA processing (Ping et al., 2014). Similarly, KO of METTL3 affects exon skipping and intron retention in mouse embryonic stem cells (mESCs; Geula et al., 2015). In addition, previous studies also found that METTL3 and the eraser ALKBH5 regulate the AS of mRNAs in spermatogenesis (Zhen et al., 2013; Xu et al., 2017). The demethylase FTO promotes exon skipping by preventing the recruitment of splicing factor 2 (SRSF2) to the splicing sites in 3T3-L1 preadipocytes (Zhao et al., 2014). m^6^A alters the local RNA structure and therefore affects the binding of HNRNP family proteins HNRNPC/G to m^6^A modified mRNA, thus affecting mRNAs AS (Liu et al., 2015, 2017). Additionally, a recent study reported that KO of HNRNPA2B1 and METTL3 depletion caused similar AS effects (Alarcon et al., 2015a). A latest study by Liu et al. (2020) suggested that YTHDC1 can facilitate the degradation of m^6^A-modified carRNAs, such as LINE1 elements, through the NEXT complex in nucleus. Notably, the m^6^A nuclear reader YTHDC1 regulates mRNA splicing by recruiting the serine/arginine-rich splicing factors SRSF3 (exon retention) and SRSF10 (exon excision; Xiao et al., 2016; Figure 2).

Several studies illustrated that transcripts with m^6^A modifications more often had proximal APA sites, while unmethylated transcripts tended to use distal APA sites (Ke et al., 2015; Molinie et al., 2016). VIRMA preferentially targets m^6^A mRNA methylation in stop codons and the 3′UTR, and it interacts with the polyadenylation cleavage factors F5 and CPSF6, thus regulating APA (Yue et al., 2018). In addition, YTHDC1 also promotes the export of m^6^A-modified mRNAs by interacting with pre-mRNA 3′ end processing factors CPSF6, splicing factors SRSF3/7, and nuclear RNA export factor 1 (NXF1; Roundtree et al., 2017b), suggesting a similar function of YTHDC1 with VIRMA in regulating APA. Moreover, a latest study illustrated that a novel m^6^A reader, FMRP, could promote the nuclear export of m^6^A-modified mRNAs through another nuclear RNA export factor, CRM1, during neural differentiation (Edens et al., 2019; Figure 2).

#### m^6^A Affects mRNA Decay, Stabilization and Translation

Transcripts transported into the cytoplasm are recognized and regulated by a series of cytoplasmic m^6^A readers, such as YTHDFs, IGF2BPs, YTHDC2, and EIF3a. The cytoplasmic m^6^A reader YTHDF2 regulates mRNA decay by recruiting the CCR4-NOT deadenylase complex, which accelerates RNA deadenylation, and degradation of m^6^A-modified mRNAs (Du et al., 2016). In contrast to YTHDF2, the cytoplasmic m^6^A readers IGF2BPs enhance mRNA stability and facilitate translation of m^6^A-modified mRNAs (Huang et al., 2018). YTHDF1 and YTHDF3 synergistically recruit translation initiation factors to promote mRNA translation and affect YTHDF2-mediated degradation of m^6^A-modified mRNAs (Wang et al., 2015; Li et al., 2017). As the only helicase-containing reader, YTHDC2 can resolve mRNA secondary structures and promote translation elongation of mRNA with m^6^A modification in the CDS region (Mao et al., 2019). Unexpectedly, cytoplasmic METTL3 could interact with eIF3h to enhance translation, suggesting that METTL3 might directly regulate translation in a m^6^A reader-independent manner (Lin et al., 2016; Choe et al., 2018). Under heat stress conditions, YTHDF2 changes subcellular localization, moving from the cytosol to the nucleus, and it preserves the level of 5′UTR m^6^A in mRNAs, thus promoting cap-independent translation (Zhou et al., 2015). In addition, m^6^A in the 5′UTR could promote cap-independent translation of mRNAs by binding with eIF3a under stress conditions (Meyer et al., 2015; Figure 2).

Together with the role that YTHDF1, YTHDF3, and YTHDC2 play in translation, YTHDF2 and IGF2BPs play crucial roles in maintaining the RNA metabolic balance in different physiological states. In addition, it is important to point out that non-coding RNAs are also regulated by RNA m^6^A modification (Alarcon et al., 2015a, b; Patil et al., 2016). The m^6^A modification not only affects the cleavage, transport, stability and degradation processes of non-coding RNA but also regulates the function of biological cells by affecting the expression of non-coding RNA (Alarcon et al., 2015a, b; Yang et al., 2017; Zhou et al., 2017). In some cases, these non-coding RNAs affect the RNA-RNA or RNA-protein interactions to regulate specific biological functions. The variability of m^6^A reader subcellular localization, the preferences across various gene regions and consensus sequences adds layers of complexity to the m^6^A regulation mechanism.

## Role of m^6^A in Stem Cell Fate

Mutipotent stem cells have been widely applied in regenerative medicine, disease treatment and organ transplantation. It has been found that RNA m^6^A modification plays essential roles in stem cell self-renewal, differentiation, and cell reprogramming (Batista et al., 2014; Wang Y. et al., 2014; Aguilo et al., 2015; Chen et al., 2015; Geula et al., 2015). It seems that m^6^A modifications have distinct effects on the fate of stem cells at various stages, states and types of stem cells.

### Role of m^6^A in Pluripotent Stem Cell Fate Decisions

It is believed that the establishment, maintenance and transition of the cell states are regulated by a series of molecular regulation mechanisms. Epigenetic regulation, such as DNA modification, histone modification, chromatin remodeling, and the work of non-coding RNAs, play significant roles in early embryonic development and the self-renewal and directional differentiation of stem cells (Kouzarides, 2007; Smith and Meissner, 2013; Bao et al., 2015). Embryonic stem cells (ESCs) derived from the inner cell mass (ICM) of blastocysts are described as being in a naïve state. As a class of versatile stem cell, naïve ESCs but not primed ESCs have the ability to form chimeric embryos. Primed epiblast stem cells (EpiSCs) derived from the ICM-derived epiblast of postimplantation embryos, represent a type of ESCs in a differentiated state. Induced pluripotent stem cells (iPSCs) were originally obtained by introducing four exogenous transcription factors (KLF4, OCT4, c-MYC, and SOX2) into the somatic cells with a virus (Maherali et al., 2007), and they have morphological and epigenetic characteristics similar to those of ESCs. Scientists have studied the molecular regulatory mechanism by which ESCs maintain self-renewal and trigger differentiation for a long time. DNA methylation, histone methylation, histone acetylation modification and non-coding RNAs have been found to contribute to determining the fate of pluripotent stem cells (Azuara et al., 2006; Doi et al., 2009; Durruthy-Durruthy et al., 2016). Recent studies found that RNA m^6^A modification might play crucial roles in pluripotent stem cell self-renewal and differentiation to specific lineages (Batista et al., 2014; Wang Y. et al., 2014; Aguilo et al., 2015).

**FIGURE 3:**
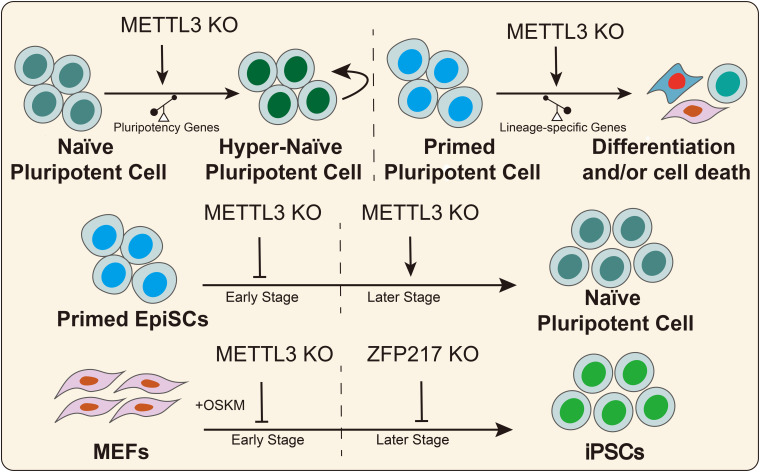
m^6^A regulates the pluripotent stem cell fate. METTL3 or m^6^A deletion has divergent effects on the fate decision of pluripotent stem cells in different states. METTL3 and ZFP217 play divergent roles in somatic cell reprogramming at different stages.

In 2014, Batista et al. (2014) found that the RNA of core pluripotency transcription factors had m^6^A modifications that were conserved in mouse and human ESCs. Wang Y. et al. (2014) illustrated that knockdown of the methyltransferases METTL3 and METTL14 inhibited the expression of pluripotency genes, such as SOX2, NANOG, and DPPA3, and it promoted the expression of developmental regulators, such as FGF5, CDX2, and SOX17 in mouse ESCs. Furthermore, METTL3 and METTL14 deletion increased RNA stability in a HuR- and miRNA-dependent manner in mESCs (Wang Y. et al., 2014). Subsequently, another study reported that Zc3h13 interacts with WTAP, VIRMA, and CBLL1, forming a biochemical complex in mESCs (Wen et al., 2018). Zc3h13 deletion decreases the global m^6^A mRNA modification levels and the ability to self-renewal, triggering the differentiation of mESCs (Wen et al., 2018). In addition, Aguilo et al. (2015) reported that zinc finger protein 217 (ZFP217) was a direct regulator of the pluripotency genes (NANOG, SOX2, KLF4, and c-MYC) in mESCs. On the other hand, ZFP217 interacts with METTL3, thus promoting mESCs self-renewal by decreasing m^6^A RNA modification of pluripotency factors and maintaining their expression levels (Aguilo et al., 2015). Similarly, a recent study revealed that METTL3 depletion prevents self-renewal of porcine iPSCs and triggers differentiation by inactivating the JAK2-STAT3 pathway in an m^6^A-YTHDF1/YTHDF2-dependent manner (Wu et al., 2019c). Conversely, several studies suggested that METTL3 deletion or m^6^A loss prolonged NANOG expression, thus promoting mESCs self-renewal and blocking differentiation (Geula et al., 2015). These seemingly contradictory outcomes could be explained by the hypothesis that m^6^A modification affects stem cell fate decisions by regulating the predominant genes in stem cells that possess different pluripotency states. In naïve ESCs, METTL3 knockdown or m^6^A loss further increased the expression of high abundance-pluripotency genes thus creating hypernaïve pluripotent states, despite the weak upregulation of lineage-specific regulators. For EpiSCs, m^6^A loss promotes the expression of lineage-specific genes, thus triggering cell differentiation (Figure 3).

Embryonic stem cell-specific lncRNAs contribute to the pluripotency maintenance (Guttman et al., 2011). Recently, Yang et al. (2018) reported that the m^6^A modification deposited on the mESC-specific lincRNA linc1281 affected mESC differentiation by regulating the linc1281-Let-7 family-Lin28 ceRNA pathway. Further, the TGFβ-Activin-Nodal signaling pathway plays crucial roles in pluripotent stem cell fate decisions. In human ESCs, Activin and Nodal activate SMAD2/3, which binds with NANOG, thus maintaining cell self-renewal. A recent study found that SMAD2/3 promotes m^6^A deposition onto nascent transcripts by recruiting the MTC, thus facilitating exit from pluripotency toward lineage-specific differentiation in human ESCs (Bertero et al., 2018).

### Role of m^6^A in Cell Reprogramming

Increasing evidence has indicated that regulating epigenetic modifications could increase somatic cell reprogramming efficiency (Zhang J. et al., 2019). Recent studies suggested that RNA m^6^A modifications can act as a functional regulator in cell reprogramming (Bao et al., 2015; Figure 3). In 2015, Geula et al. (2015) reported that METTL3 deletion suppressed mouse EpiSC reprogramming efficiency toward naïve pluripotency in early stages but had the opposite effect in the later stages. In MEFs, early KO of METTL3 diminished somatic reprogramming efficiency but did not affect reprogramming efficiency after 3 days of induction with OKSM factors (Geula et al., 2015). Subsequently, Chen et al. (2015) reported that METTL3 overexpression increases m^6^A abundance and promoted the expression of pluripotent factors and colony numbers of iPSCs, suggesting that m^6^A promotes cell reprogramming. Another study suggested that the expression level of ZFP217 gradually increased during somatic reprogramming, while METTL3 exhibited the opposite trend (Aguilo et al., 2015). ZFP217 deletion diminished the number of colonies positive for alkaline phosphatase, while ZFP217 overexpression promoted the formation of iPSC colonies (Aguilo et al., 2015). Furthermore, METTL3 knockdown partially rescued somatic cell reprogramming in Zfp217-depleted cells. Mechanistically, ZFP217 transcription activates core reprogramming factors, thus maintaining cell stemness. On the other hand, ZFP217 binds with METTL3, thus diminishing m^6^A deposition onto the transcripts of pluripotency genes. Taken together, these studies illustrated the important and various functional regulatory roles of ZFP217 and METTL3 in somatic cell reprogramming. METTL3 is essential for somatic cell reprogramming because it arrests the cell cycle, thus affecting cell proliferation in the early stage, while ZFP217 is indispensable for activating pluripotency factors in the later stages of somatic cell reprogramming.

### Role of m^6^A in the Differentiation of Other Stem and Progenitor Cells

Several studies have suggested that m^6^A RNA modification plays crucial roles in the hematopoietic system, neural system, fat metabolism and muscle development. Mesenchymal stem cells (MSCs) derived from different tissues may be suitable for the treatment of various diseases due to the different secretory capacities of cytokines and growth factors.

**FIGURE 4:**
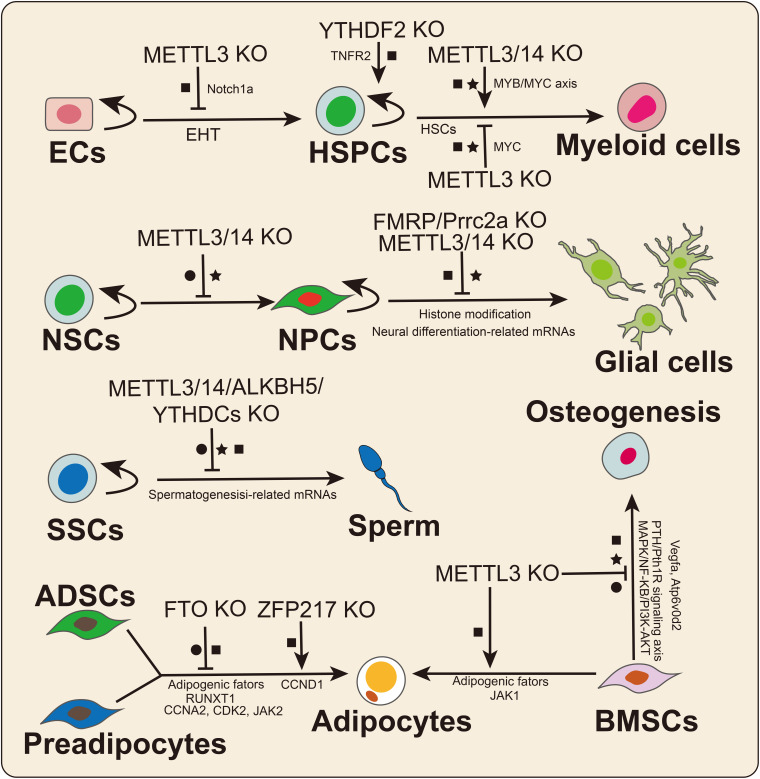
The crucial roles of m^6^A in stem cell and progenitor cell differentiation. m^6^A plays divergent functions by affecting mRNA splicing (•), stability (■), and translation (★). ECs: endothelial cells; HSPCs: hematopoietic stem/progenitor cells; NSCs: neural stem cells; NPCs: neural progenitor cells; SSCs: spermatogonial stem cells; ADSCs: adipose-derived stem cell; and BMSCs: bone marrow mesenchymal stem cells.

In 2017, Zhang et al. (2017) found that m^6^A determines the hematopoietic stem/progenitor cell (HSPC) fate by affecting the endothelial-to-hematopoietic transition (EHT) during zebrafish embryogenesis (Figure 4). Furthermore, METTL3 deletion downregulated the m^6^A modification level of Notch1a transcripts, thus protecting the mRNA from YTHDF2-mediated decay to block EHT during vertebrate embryogenesis (Zhang et al., 2017). Subsequently, Vu et al. (2017) reported that METTL3 deletion promotes human HSPC differentiation and inhibits cell proliferation. Mechanistically, the knockdown of METTL3 hindered C-MYC, BCL-2, and PTEN translation and stimulated AKT to be phosphorylated to trigger downstream signaling pathway reactions (Vu et al., 2017). METTL14 was shown to be highly expressed in normal HSPCs and downregulated during myeloid differentiation (Weng et al., 2018). Similarly, METTL14 knockdown promoted terminal myeloid differentiation of normal HSPCs by inactivating the MYB/MYC axis. In contrast, Lee et al. (2019) reported that METTL3 deletion inhibited the differentiation of hematopoietic stem cells (HSCs) but led to the accumulation of HSCs in adult bone marrow. Yao et al. (2018) also confirmed that METTL3/14 played essential roles in maintaining the self-renewal of HSCs in adult bone marrow. Similarly, Cheng et al. (2019) also found that METTL3 maintained the symmetric commitment and identity of HSCs by affecting MYC mRNA stability. The divergent effects of m^6^A on HSC fate may be because m^6^A plays different roles in HSC and progenitor fate determination. Notably, abnormal expression of METTL3/14 may lead to the occurrence of malignant diseases related to the hematopoietic system, such as acute myeloid leukemia (AML; Vu et al., 2017; Weng et al., 2018). In addition, two recent studies illustrated that the m^6^A reader YTHDF2 was required for HSC self-renewal and AML initiation by mediating mRNA degradation (Li et al., 2018; Paris et al., 2019). YTHDF2 deletion not only promoted HSC expansion but also prevented leukemia initiation, suggesting that YTHDF2 could be a potential therapeutic target in AML (Paris et al., 2019).

Spermatogonial stem cells (SSCs) are a class of stem cells that not only can self-renew but also can differentiate into spermatocytes. Spermatogenesis involves a highly regulated differentiation process that includes mitosis, meiosis, and spermiogenesis. Multiple studies have suggested that KO of the m^6^A effector has significant effects on male fertility and spermatogenesis. In 2017, Lin et al. (2017) first investigated the dynamic changes in m^6^A levels during spermatogenesis, and m^6^A was found to be relatively highly enriched in pachytene spermatocytes and round spermatids. Germ cell-specific METTL3 or METTL14 KO caused mRNA translation dysregulation, thus affecting SSC proliferation and differentiation (Lin et al., 2017). Similarly, another study reported that germ cell-specific METTL3 deletion prevented spermatogonial differentiation and meiosis by altering transcript expression and splicing (Xu et al., 2017). Moreover, other studies suggested that ALKBH5, YTHDC1, and YTHDC2 KO mice exhibited deficient phenotypes in spermatogenesis or male fertility (Zhen et al., 2013; Kasowitz et al., 2018), suggesting crucial roles for m^6^A in spermatogenesis and animal reproduction. Further studies regarding the regulatory mechanisms of m^6^A on spermatogenesis or male fertility need to be performed.

RNA m^6^A modification is indispensable for the development and functional maintenance of the nervous system. METTL3 and METTL14 deletion prolonged the cell cycle progression of cortical neural progenitor cells (NPCs) and reduced the differentiation of radial glial cells (RGCs) during mouse embryonic cortical neurogenesis (Yoon et al., 2017). Oligodendrocyte-specific METT14 deletion resulted in myelin abnormalities and decreased oligodendrocyte numbers but did not affect oligodendrocyte precursor cell (OPC) numbers. In OPCs, METTL14 ablation prevented the differentiation of OPCs, suggesting that m^6^A is a crucial regulator of oligodendrocyte differentiation. The epitranscriptome analysis results suggested that a large number of transcripts related to oligodendrocyte lineage progression were marked with m^6^A modification. METTL14 deletion led to aberrant mRNA splicing in OPCs as well as oligodendrocytes. Wang et al. (2018) reported that METTL14 deletion decreased cell proliferation and promoted untimely differentiation of eNSCs. Moreover, METTL14 deletion increased H3K4me3, H3K27me3, and H3K27ac levels by affecting the mRNA stabilization of histone-modifying enzymes in eNSCs (Wang et al., 2018). In contrast, a recent study found that METTL3 deletion reduced the level of histone methyltransferase Ezh2, thereby decreasing H3K27me3 levels in adult neural stem cells (aNSCs; Chen et al., 2019). Mechanistically, KO of METTL3 inhibited the proliferation of aNSCs and promoted aNSC differentiation toward the glial lineage. In addition, EZH2 overexpression rescued the defects resulting from METTL3 depletion. The diverse effects of m^6^A may be due to differences in methyltransferase functions and cell types. Notably, recent studies have identified two new neuronal cell-specific m^6^A readers, FMRP and Prcc2a, that play important roles in nervous system development in mice. FMRP deletion hindered cycle progression and promoted the proliferation of NPCs, which was similar to what was observed in METTL14 conditional KO mice (Edens et al., 2019). Mechanistically, FMRP promotes m^6^A-modified mRNA nuclear export through CRM1 during neural differentiation (Edens et al., 2019). Another neuronal cell-specific m^6^A reader is Prrc2a. In mice, Prrc2a regulates OPC proliferation and oligodendrocyte fate by stabilizing Olig2 mRNA during oligodendrocyte development (Wu et al., 2019b).

Marrow MSCs are a class of stem cells derived from marrow, adipose and umbilical cord tissues, and they can be differentiated into osteoblasts, chondrocytes, and adipocytes. Among MSCs, bone marrow mesenchymal stem cells (BMSCs) have been applied to the cell-based therapy for some osteoporosis-related and human cancers. In 2018, Wu Y. et al. (2018) identified that MSC-specific METTL3 deletion inhibited osteogenic differentiation while promoting adipogenic differentiation *in vivo* and *in vitro*. The translation efficiency of parathyroid hormone receptor-1 (Pth1r) was impaired in METTL3-deficient MSCs. In contrast, overexpression of METTL3 could rescue osteoporosis in mice (Wu Y. et al., 2018). Mechanistically, METTL3 deletion regulated osteogenesis by affecting the PTH/Pth1r signaling axis in an m^6^A-dependent manner. Subsequently, Tian et al. (2019) found that the expression level of METTL3 was significantly increased during osteogenic differentiation of BMSCs. Knockdown of METTL3 inhibited osteogenic differentiation, reduced p-AKT levels and affected the expression of genes in PI3K-AKT signaling pathways; the knockdown also affected the AS of VEGFa (Tian et al., 2019). Similarly, a recent study demonstrated that METTL3 regulated osteoclast differentiation by protecting the Atp6v0d2 mRNA from the degradation by YTHDF2 and promoted the nuclear retention of Traf6 mRNA (Li D. et al., 2020). METTL3 deletion prevented the expression of osteoclast-specific genes, decreased the phosphorylation levels of key factors in the MAPK, NF-κB, and PI3K-AKT signaling pathways and inhibited osteoclast differentiation (Li D. et al., 2020). A recent study revealed that the expression level of METTL3 was significantly increased during adipogenesis in porcine BMSCs (Yao et al., 2019). Moreover, METTL3 deletion promoted adipogenesis by activating the JAK1/STAT5/C/EBPβ pathway in an m^6^A-YTHDF2-dependent manner in porcine BMSCs (Yao et al., 2019). These findings suggested that m^6^A could be a crucial link between adipogenic and osteogenic lineages and that METTL3 might be a potential treatment target for the osteoporosis. Increasing evidence has also found that m^6^A regulates adipose-derived stem cell (ADSC) or preadipocyte fate decisions (Zhao et al., 2014; Wu R. et al., 2018; Liu Q. et al., 2019; Wu et al., 2019a).

In 3T3-L1 preadipocytes, FTO regulates adipogenesis by affecting SRSF2 binding with m^6^A-modified mRNAs thus leading to exon skipping (Zhao et al., 2014). On the other hand, FTO regulated the transcript abundance of cell cycle-related genes in an m^6^A-YTHDF2-dependent manner in 3T3-L1 preadipocytes (Wu R. et al., 2018). In addition, FTO promotes adipogenesis by decreasing the m^6^A level of JAK2, thus protecting its mRNA from degradation by YTHDF2 and activating the JAK2-STAT3-C/EBPβ signaling pathway during adipogenic differentiation (Wu et al., 2019a). In 3T3-L1 cells, KO of ZFP217 promoted METTL3 expression and increased global RNA m^6^A levels, thus downregulating the expression level of CCND1 in an m^6^A-YTHDF2-dependent manner (Liu Q. et al., 2019). In normal 3T3-L1 cells, ZFP217 promotes adipogenesis by activating FTO and interacting with YTHDF2 (Liu Q. et al., 2019). However, KO of ZFP217 increased the global RNA m^6^A level by decreasing FTO expression in a m^6^A-YTHDF2-dependent manner (Liu Q. et al., 2019). On the other hand, ZFP217 deletion directly decreased the expression level of FTO in 3T3-L1 cells (Liu Q. et al., 2019). Taken together, ZFP217 might act as a “super” m^6^A regulator by interacting with the m^6^A writer METTL3, eraser FTO, and reader (YTHDF2), thus playing functional roles in various cellular physiological processes.

## m^6^A Modification and Mammalian Embryonic Development

**FIGURE 5:**
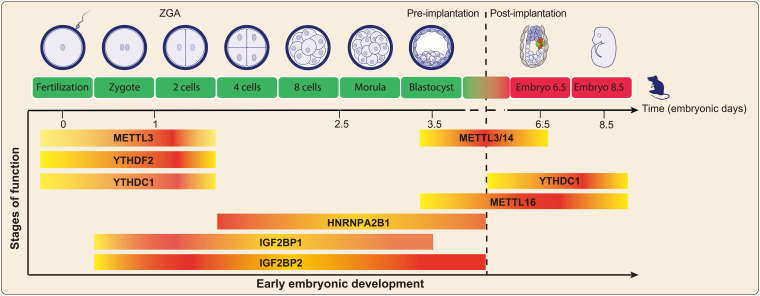
The m^6^A modification-related proteins exert essential functions in early embryonic development. The highlighted color indicates that the proteins mainly play functions at this stage.

As one of the most important molecular processes in life, early mammalian embryonic development is determined by multiple cell fate decisions that result in an overall development blueprint for organogenesis and morphogenesis. The process of early embryonic development involves the maintenance and differentiation of totipotent cells, which is determined by the order of differentiation of various pluripotent stem cells. Embryo development is known to be regulated by a series of complex regulatory mechanisms at different levels. An increasing number of studies have suggested that RNA m^6^A modification is associated with animal reproduction programs, such as gametogenesis, maternal-zygote transition, and early embryo development (Zhen et al., 2013; Geula et al., 2015; Du et al., 2016; Zhao et al., 2017; Mendel et al., 2018; Sui et al., 2020; Figure 5).

### The Influences of m^6^A on Preimplantation Embryo Development

The maternal-to-zygotic transition (MZT) is one of the most important highly ordered and regulated events during early embryo development. The MZT is accompanied by the degradation of maternal RNA and protein and zygote genome activation (ZGA). As animal embryonic development proceeds, posttranscriptional mechanisms act as critical regulators to ensure suitable gene expression and timely progression of development programs.

In 2015, Geula et al. (2015) illustrated that METTL3 KO blastocysts of mouse showed normal morphology and normal expression of pluripotency markers (OCT4 and NANOG) compared with wild type blastocysts at E3.5 (embryonic day E3.5), suggesting that METTL3 might not affect preimplantation embryonic development of mouse. Notably, a recent study first detected the RNA m^6^A level during mouse oocyte maturation and embryonic development by immunofluorescence (IF) staining (Sui et al., 2020). The global RNA m^6^A level was gradually decreased from the germinal vesicle (GV) to the two-cell stage but increased after the two-cell stage during preimplantation embryos development (Sui et al., 2020). Knockdown of METTL3 caused an approximately half of oocytes spindle abnormalities, and the proportion of aneuploid oocytes rose, thereby hindering oocytes maturation in mice (Sui et al., 2020). In addition, METTL3 deletion decreased mRNA translation efficiency in oocytes. In parthenogenetically activated embryos, METTL3 deletion impeded the mouse ZGA process by affecting maternal mRNA degradation, and the results of EU (5-Ethynyl uridine) and phosphor-pol II ser2/5 staining also suggested that METTL3 deletion reduced the global transcription level in the two-cell embryos of mouse (Sui et al., 2020). Subsequently, Kwon et al. (2019) found that the m^6^A reader hnRNPA2B1 was regulated by METTL3 and played a crucial role in mouse embryonic development. In mice, HNRNPA2B1 is highly expressed in embryos at the 4-cell stage (Kwon et al., 2019). Knockdown of HNRNPA2B1 downregulated the expression of pluripotency-related genes, such as OCT4, and SOX2. METTL3 deletion decreased the mean sizes of blastocysts and the proportion that reached the morula and blastocyst stage (Kwon et al., 2019), suggesting that HNRNPA2B1 contributes to blastocyst quality. Moreover, METTL3 deletion increased the mislocalization of HNRNPA2B1 in blastocysts and resulted in embryonic developmental defects, which was similar to the effect observed in blastocysts following hnRNPA2B1knockdown (Kwon et al., 2019).

In 2017, two studies illustrated that the m^6^A reader YTHDF2 is required for oocyte maturation and early embryonic development (Ivanova et al., 2017; Zhao et al., 2017). Mechanistically, YTHDF2 recognizes m^6^A-modified mRNAs and mediates the m^6^A-dependent RNA degradation process during mouse ZGA (Ivanova et al., 2017). YTHDF2 deletion delayed the degradation of maternal mRNAs, thus impeding zygotic genome activation in mice. These results indicate that YTHDF2-mediated m^6^A-dependent mRNA degradation plays an important role in transcriptome transitions and early embryonic development. Similar to the YTHDF2 knockout mouse model, a recent study found that oocyte-specific deletion of VIRMA results in female-specific infertility in mice (Yue et al., 2018). VIRMA deletion affected oocyte meiotic maturation by regulating pre-mRNA AS (Yue et al., 2018). The role of VIRMA in early embryonic development needs to be investigated in the future.

In contrast to YTHDF2, which functions in the promotion of mRNA decay, recent studies revealed that IGF2BPs could act as a new class of cytoplasmic m^6^A readers that promote the stability and storage of mRNAs (Huang et al., 2018). It was reported that IGF2BP1 deletion suppressed the RNA m^6^A modification level in mouse embryos and caused impaired early parthenogenetic (PA) embryogenesis, whereas supplementation with betaine increased the RNA m^6^A modification level and rescued embryonic development (Hao et al., 2020). In addition, miR-670-3p was found to regulate IGF2BP1 and affect apoptosis in PA embryos (Hao et al., 2020). Subsequently, Liu H.B. et al. (2019) reported that IGF2BP2 (also known as IMP2) is highly expressed in oocytes and early-stage embryos. IGF2BP2 deletion was not essential for oocyte maturation, while IGF2BP2-knockout mice exhibited female infertility (Liu H.B. et al., 2019). Moreover, maternal deletion of IGF2BP2 caused embryos to arrest at the 2-cell stage (Liu H.B. et al., 2019). Transcriptome and proteome analyses suggested that knockout of IGF2BP2 inhibited the transcription and translation of downstream genes related to ZGA, such as CCAR1 and RPS14 (Liu H.B. et al., 2019). However, whether IGF2BP2 participates in regulation of mRNA stability in ZGA as an m^6^A reader still needs to be clarified. Notably, a recent study found that IGF2BP3, but not IGF2BP1/2, stabilized maternal mRNA and regulated early embryogenesis in zebrafish (Ren et al., 2020). The majority of target mRNAs of IGF2BP3 were different from those of YTHDF2, suggesting that IGF2BPs and YTHDF2 may be different, but indispensable regulators at the MZT stage of early embryonic development in various species.

### The Influences of m^6^A on Postimplantation Embryo Development

After the embryo is implanted in the uterus, cells in the ICM transition from naïve state pluripotency to primed state pluripotency, and the blastocyst differentiates into the epiblast of the embryonic region. The ectoderm, mesoderm and endoderm, which are derived from the epidermal cells through the gastrulation, constitute the basic cells required for organogenesis and individual formation.

Consistent with the observation that m^6^A is indispensable for ESC fate decisions, the loss of m^6^A causes naïve pluripotent stem cells to enter into a “hyper” naïve state, and they cannot transition from naïve state toward lineage differentiation, thus leading to embryonic lethality (Geula et al., 2015). *In vivo*, METTL3^–/–^ ESCs showed a poor ability to differentiate into mature teratomas, and hematoxylin-eosin staining (H&E) staining suggested that KO teratomas could not differentiate into the three germ layers (Geula et al., 2015). The abnormal expression and location of NANOG from E5.5 (early postimplantation) to E7.5 (late gastrointestinal motility) in epiblasts led to embryonic lethality (Geula et al., 2015). MELLT3 and METTL14 knockout (KO) mice both exhibited embryonic lethality at E6.5 (Geula et al., 2015; Meng et al., 2019), suggesting that METTL3/14 are indispensable for early embryonic development in mice. A recent study reported that METTL14 deletion results in a similar effect to that of METTL3 in mouse embryogenesis. Compared with E5.5 WT embryos, Mettl14^–/–^ embryos exhibited a large difference in gene expression and AS events, especially exon skipping (Meng et al., 2019). One possible mechanism is that METTL3/14 or m^6^A deposition accelerates the conversion of the epiblast from a naïve to a primed state, thus promoting differentiation. The aberrant expression levels of naïve and primed makers in METTL14^–/–^ embryos support this inference.

A previous study found that WTAP deletion resulted in abnormal egg cylinders at the gastrulation stage and led to embryonic lethality at E10.5 in mice (Fukusumi et al., 2008). WTAP-deficient ESCs failed to differentiate into endoderm and mesoderm, which was confirmed by chimera analysis (Fukusumi et al., 2008). These results suggested that WTAP is essential for mesoderm and endoderm differentiation in the early mouse embryo. Notably, whether WTAP, as an m^6^A effector, plays a functional regulatory role in this process and the relevant regulatory mechanism has yet need to be determined.

In 2017, Pendleton et al. first reported that the m^6^A writer METTL16 regulated MAT2A expression by affecting its splicing in a hairpin (hp1) m^6^A-dependent manner (Pendleton et al., 2017). Subsequently, Mendel et al. created a METTL16 knockout (METTL16^–/–^) mouse model (Mendel et al., 2018). The E2.5 morula and E3.5 blastocysts from METTL16 KO and WT mice exhibited normal morphology and genotyping ratios. However, only a small proportion (1.9%) of KO embryos were detected at E6.5, indicating that METTL16 deletion caused embryonic lethality around implantation (Mendel et al., 2018). Interestingly, transcriptome analysis results suggested that MAT2A was the most downregulated gene in KO embryos at the E2.5 morula stage, whereas a large number of genes were dysregulated in E3.5 blastocysts of mouse (Mendel et al., 2018). Taken together, the m^6^A writer METTL16 regulates the methylation of the mRNA encoding SAM synthetase MAT2A and participates in the development of mouse embryos.

Several studies have demonstrated that the cytoplasmic reader YTHDC2 is essential for the transition from mitosis to meiosis in germ cells (Bailey et al., 2017; Hsu et al., 2017; Wojtas et al., 2017). YTHDC2 is dispensable for viability, while YTHDC2 KO mice are infertile. Similar to METTL3, deletion of YTHDC1 results in embryonic lethality of mouse (Kasowitz et al., 2018). A recent study reported that YTHDC1 not only was required for gametogenesis but was also essential for viability (Kasowitz et al., 2018). Mechanistically, as the only nuclear m^6^A reader, YTHDC1 regulates the APA, AS and nuclear export of m^6^A-modified mRNAs in mouse oocytes (Kasowitz et al., 2018). The non-redundant role of YTHDC1 is also indispensable for mouse early embryonic development. In addition, outgrowth analysis found that no colonies were formed in HNRNPA2B1 KO blastocysts, unlike WT blastocysts, suggesting that HNRNPA2B1 may contribute to postimplantation development in mice (Kwon et al., 2019).

It is believed that m^6^A modifications are essential for the embryonic development and fertility in animals, whereas the regulatory mechanism is still largely unknown. One reason is that the majority of m^6^A-related enzyme deletions lead to early embryo lethality. On the other hand, m^6^A-seq requires a large amount of RNA sample input, which restricts the application of m^6^A to early embryos. The function and molecular regulation mechanism of RNA m^6^A modification in mouse early development need to be further investigated.

## Perspectives

The precise temporal and spatial control of gene expression is of fundamental importance for establishing cell fate and early development of complex bodies. Reversible RNA m^6^A methylation has many of the same characteristics as epigenetic DNA and histone modifications. Although epigenetic DNA and histone modifications mainly affect transcription events, reversible RNA methylation mainly regulates gene expression at posttranscriptional level. The dynamic regulation of m^6^A represents a newly identified mechanism of posttranscriptional regulation that maintains the balance between pluripotency and cell differentiation in a timely manner to ensure proper development. Different physiological states, environmental stimulates and cell signaling events may trigger different m^6^A methylation functions. Notably, there exist controversy on whether distinct cytoplasmic m^6^A-binding proteins YTHDFs recognize different sites (Wang et al., 2015; Li et al., 2017; Zaccara and Jaffrey, 2020). The majority of current studies suggested that YTHDF1/3 regulate translation of m^6^A-modified mRNAs (Wang et al., 2015; Li et al., 2017; Shi et al., 2017). However, how do these m^6^A readers (YTHDFs, YTHDCs, and IGF2BPs) specifically recognize their target transcripts during stem cell fate decisions and early embryonic development? In contrast to prevailing model, Zaccara and Jaffrey proposed that DF proteins do not regulate translation in HeLa cells, and YTHDFs bind the same m^6^A-modified mRNAs rather than different mRNAs in mRNA degradation (Zaccara and Jaffrey, 2020). Notably, it needs to be re-examined on the conclusion came only by siRNA knockdown in HeLa cells. Recently, Zhang et al. (2020) proposed that the effects of m^6^A on translation are heterogeneous and context dependent. Indeed, it still needs to be investigated the complex function of YTHDFs in translation. In addition, further studies and a comprehensive understanding of the functional roles of other m^6^A effectors during early embryonic development in mammals are still needed.

As we review in this review, RNA m^6^A modification plays significant functional roles in stem cell fate decisions and early embryonic development. Given that pluripotent stem cells have great prospects for application in regenerative medicine, organ transplantation and cancer treatment, a more in-depth study of the molecular regulation mechanisms by which m^6^A regulates the fate of pluripotent and other stem cells will promote the use of stem cells in developing and instituting stem cell treatments in the clinic. A number of studies have shown that RNA m^6^A modification is essential for mammalian embryo development and fertility. Although the majority of cloned animals are viable and can be produced via somatic cell nuclear transfer (SCNT), the efficiency is still extremely low (Wilmut et al., 1997; Matoba et al., 2018). One of the most important factors impeding SCNT is that abnormal epigenetic modification in donor cells, such as DNA methylation, histone modifications, and genomic imprinting (Yamagata et al., 2007; Zhang et al., 2009; Okae et al., 2014). Notably, SCNT is accompanied by significant events, including somatic cell reprogramming, ZGA and embryonic development, which have all been revealed to be regulated by RNA m^6^A modification. It remains to be examined whether m^6^A could affect SCNT embryonic development following SCNT. Moreover, it is of great significance to improve the SCNT efficiency by regulating the m^6^A modification level in somatic cells.

It is difficult to collect large amounts of samples from patients in the clinic and early embryos, whereas m^6^A-seq technological currently requires a large amount of RNA sample (>20 μg total RNA). New or refined m^6^A-seq technologies with low amounts of RNA samples input (>500 ng total RNA) or antibody-independent methods (MAZTER-seq, m^6^A-REF-seq, DART-seq, m^6^A-SEAL, and m^6^A-label-seq) have great application potential in the study of the m^6^A epitranscriptome in relation to disease treatment and embryonic development (Zeng et al., 2018; Meyer, 2019; Garcia-Campos et al., 2019; Zhang Z. et al., 2019; Shu et al., 2020; Wang et al., 2020). Moreover, recent studies have developed m^6^A editing technologies that enable methylation or demethylation at a single site in transcripts through CRISPR-dCas9 and CRISPR-dCas13b systems (Rauch et al., 2018; Liu X. M. et al., 2019; Wilson et al., 2020; Li J. et al., 2020). We believe that m^6^A modification has great potential for application in regenerative and precision medicine, including cancer treatment, organ transplantation and reproductive development.

## Author Contributions

MZ wrote the manuscript. YZ drew the pictures. SZ and XD revised the manuscript. ZL reviewed and edited the manuscript. All authors contributed to the article and approved the submitted version.

## Conflict of Interest

The authors declare that the research was conducted in the absence of any commercial or financial relationships that could be construed as a potential conflict of interest.
