# Corrigendum: Roles of N6-Methyladenosine (m^6^A) in Stem Cell Fate Decisions and Early Embryonic Development in Mammals

**DOI:** 10.3389/fcell.2021.640806

**Published:** 2021-01-21

**Authors:** Meng Zhang, Yanhui Zhai, Sheng Zhang, Xiangpeng Dai, Ziyi Li

**Affiliations:** Key Laboratory of Organ Regeneration and Transplantation of Ministry of Education, First Hospital, Jilin University, Changchun, China

**Keywords:** N6-methyladenosine, RNA metabolism, stem cell fate, cell reprogramming, embryonic development

In the original article, there was a mistake in Figure 1 as published. The three methyl groups (CH3) on the right were not placed on the appropriate nitrogen atom of the purine ring. The corrected Figure 1 appears below.

**Figure 1:**
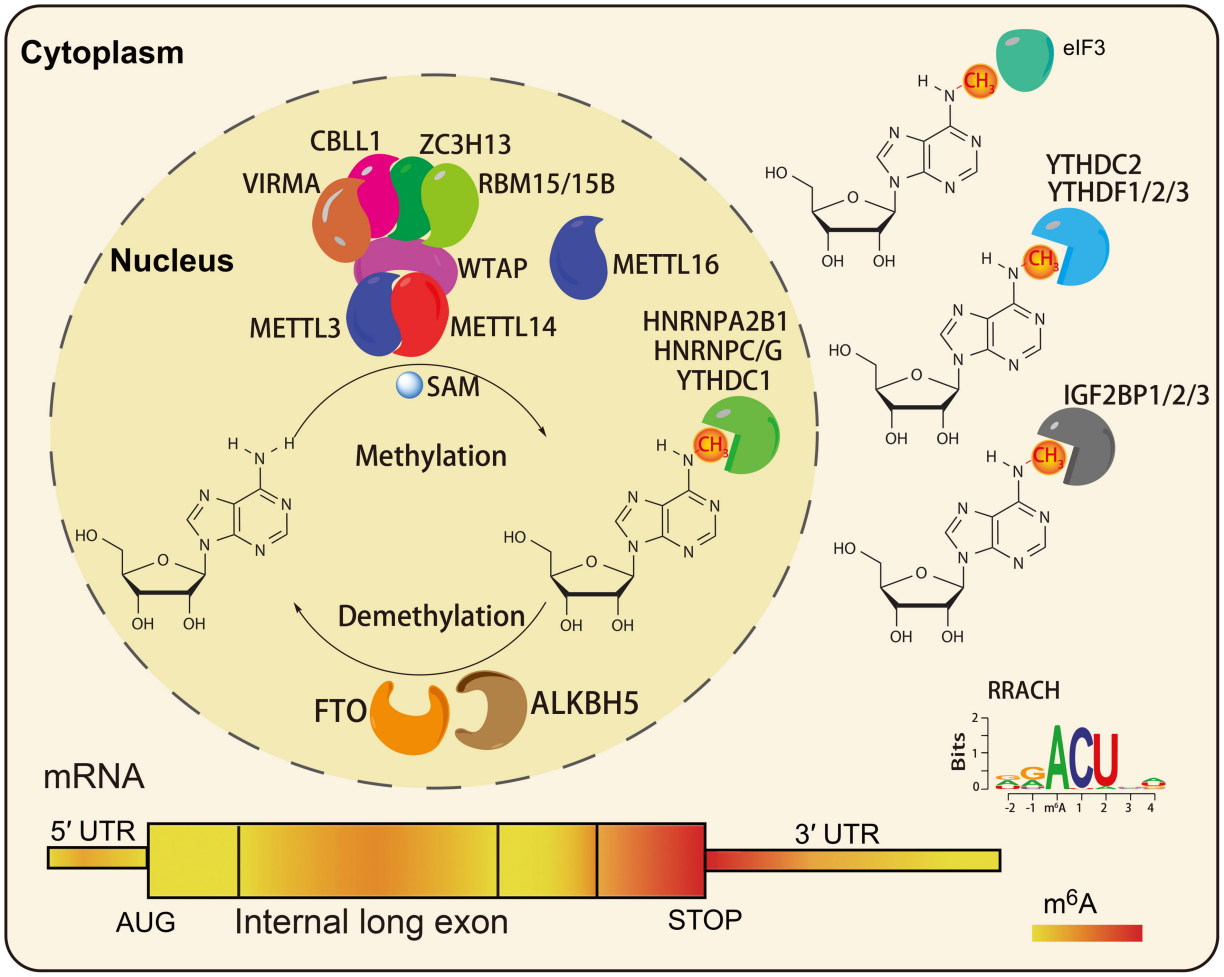
The characteristics of RNA m^6^A modification. The m^6^A writers, erasers, and readers in eukaryotic cells (Shi et al., 2019). The preferences and density of RNA m^6^A modification in different regions of mRNAs (Fitzsimmons and Batista, 2019).

The authors apologize for this error and state that this does not change the scientific conclusions of the article in any way. The original article has been updated.
